# Self-rated depression and eye diseases: The Beijing Eye Study

**DOI:** 10.1371/journal.pone.0202132

**Published:** 2018-08-10

**Authors:** Jost B. Jonas, Wen Bin Wei, Liang Xu, Marcella Rietschel, Fabian Streit, Ya Xing Wang

**Affiliations:** 1 Beijing Institute of Ophthalmology, Beijing Tongren Eye Center, Beijing Tongren Hospital, Capital Medical University; Beijing Key Laboratory of Ophthalmology and Visual Sciences, Beijing, China; 2 Department of Ophthalmology, Medical Faculty Mannheim of the Ruprecht-Karls-University, Mannheim, Germany; 3 Beijing Tongren Eye Center, Beijing Tongren Hospital, Capital Medical University, Beijing, China; 4 Department of Genetic Epidemiology in Psychiatry, Central Institute of Mental Health, Medical Faculty Mannheim, University of Heidelberg, Mannheim, Germany; University of Illinois at Chicago, UNITED STATES

## Abstract

**Purpose:**

To assess the prevalence of depression in the general population of Beijing and its association with ocular diseases.

**Methods:**

The population-based Beijing Eye Study was conducted in a rural and an urban region of Greater Beijing. The study participants underwent a detailed ophthalmological examination and an interview including questions on the socioeconomic background. Depressive symptoms were evaluated using a Chinese depression scale adapted from Zung´s self-rated depression scale. The total score of depression symptoms was 80. Depression was defined as having a depression score >44.

**Results:**

Out of 3468 study participants, 3267 (94.2%) individuals (1419 men) with an age of 64.5±9.7 years (range: 50–93 years) participated in the interview and answered all questions on depression. The mean depression score was 25.0±5.9 (median: 23.3; range:20–64). Depression (depression score >44) was present in 66 individuals (2.0%; 95% confidence interval (CI): 1.5, 2.5), and 5 individuals (0.2%; 95%CI: 0.02,0.3) had a depression score ≥59. In multivariate regression, analysis, a higher depression score was associated (regression coefficient r^2^: 0.22) with a higher number of days with dry eye feeling (*P*<0.001; standardized regression coefficient beta: 0.09; non-standardized regression coefficient B: 0.20; 95%CI: 0.12,0.29) and shorter corneal curvature radius (*P* = 0.03;beta:-0.04; B:1.01; 95%CI: -1.90,-0.12), after adjusting for age, gender, region of habitation, body mass index, cognitive function score, life quality score and blood concentration of triglycerides. Adding age-related macular degeneration (*P* = 0.10), glaucoma (*P* = 0.77), diabetic retinopathy (*P* = 0.77), nuclear cataract (*P* = 0.35), cortical cataract (*P* = 0.58) or posterior subcapsular cataract (*P* = 0.28) as single parameters to the model revealed no significant correlation with the depression score. Lower best corrected visual acuity showed a marginal significant association (*P* = 0.05; beta: 0.04; B: 1.56; 95%CI: -0.01, 3.13).

**Conclusions:**

Dry eye feeling was the only common ocular disorder associated with an increased depression score, while the occurrence of age-related macular degeneration, any type of glaucoma, diabetic retinopathy, any type of cataract and keratoconus were not significantly associated with an increased depression score. Lower visual acuity was marginally associated. The prevalence of depression in the population aged 50+ years in Greater Beijing was 2.0% (96%CI: 1.5, 2.5).

## Introduction

Mental health disorders including depression have become main factors in the global burden of disease, since they have a relatively high prevalence, often take a chronic course, and occur at a relatively young age. They caused 5.4% of all DALYs (Disability-Adjusted Life Years) and 17.4% of all YLDs (Years Lived with Disability) in the year 2013 [1]. Since depression as one of the major mental illnesses has a profound influence on life style, socioeconomic background and personal health care, it can indirectly influence the occurrence and course of other diseases. As a corollary, decreasing quality of life due to non-mental health diseases may trigger the development of depression. Since associations between depression and ocular disorders have not systematically been analyzed yet, we conducted this study to assess the relationships between self-rated depression and major ocular diseases. The examinations included the assessment of dry eye syndrome, which was in previous investigations associated with depression [2–7]. Correspondingly, a recent study by Mrugacz and colleagues revealed that the tear fluid levels of interleukin (IL)-6, IL-17 and tumor necrosis factor (TNF)-α were significantly higher in patients with depression than in controls [7]. In a parallel manner, the clinical severity of dry eye was associated with the concentrations of IL-17 and TNF-α in tears. It led to the assumption of a pathogenetic role of the inflammatory cytokines IL-17 and TNF-α in the etiology of dry eye disease in depressive patients. This and other examples may underlie the potential associations between depression and ophthalmological disorders, the assessment of which was the purpose of this study.

## Methods

The Beijing Eye Study 2011 is a population-based cross-sectional study that was conducted in a rural region and an urban region of Greater Beijing. Its protocol was approved by the Medical Ethics Committee of Beijing Tongren Hospital, which confirmed that all methods were performed in accordance with the relevant guidelines and regulations. All study participants signed an informed consent. The only eligibility criterion for inclusion into the study was an age of 50+ years. Out of 4403 eligible individuals, 3468 subjects (1963 (56.6%) women) participated (response rate: 78.8%). The mean age was 64.6 ± 9.8 years (median: 64 years; range: 50–93 years). The study population and the study design have been described in detail previously [8,9].

All study participants underwent a structured interview by trained research technicians. The interview included standardized questions on demographic parameters, socioeconomic background, diet, alcohol consumption, smoking, and known major systemic diseases. The cognitive function was assessed applying the Mini–Mental State Examination (MMSE) test. Fasting blood samples were collected to measure the blood concentrations of lipids, glucose, glycosylated hemoglobin HbA1c and serum creatinine. Blood pressure was measured with the participants sitting for at least 5 minutes. We also determined body height and weight and the circumference of waist and hip. The ophthalmological examination consisted of an automatic refractometry (Auto Refractometer AR-610; Nidek Co., Ltd, Tokyo, Japan), measurements of presenting visual acuity, uncorrected visual acuity and best-corrected visual acuity, tonometry, slit-lamp based biomicroscopy of the anterior and posterior segments of the eyes, and photography of the cornea and lens (Neitz CT-R camera; Neitz Instruments Co., Tokyo, Japan) and of the macula and optic disc (fundus camera; type CR6-45MM; Canon Inc., Tokyo, Japan) in medical mydriasis. Using the photographs, we measured the dimensions of the optic disc, optic cup and parapapillary alpha, beta and gamma zones. The optic nerve head and macula were additionally examined by spectral-domain optical coherence tomography (OCT) using the enhanced depth imaging modality (Spectralis; Heidelberg Engineering, Heidelberg, Germany). We determined the thickness of the peripapillary retinal nerve fiber layer, of the retina in the foveal region and of the subfoveal choroid. Applying optical low-coherence reflectometry (Lenstar 900 Optical Biometer; Haag-Streit, Koeniz, Switzerland), we measured ocular biometric parameters such as the anterior corneal curvature, central corneal thickness, anterior chamber depth, lens thickness and axial length.

The subjective symptoms of dry eye were evaluated using a questionnaire composed of three questions: “Do your eyes ever feel dry?”; “Do you ever feel a gritty or sandy sensation in your eyes?”; and “Do your eyes ever have a burning sensation?” Possible answers to the questions were none (0), less than once a month (1), once or twice a week (2), at least once every day (3), or all the time (4). The presence of dry eye symptoms was defined as having one or more symptoms at least once every day (scores of 3 and 4). A quantitative grading score for subjective dry eye symptoms was obtained by summarizing the answers resulting in a total score ranging between 0 and 12. Using the lens photographs, the degree of nuclear opacities was assessed in 6 grades using the grading system of the Age-Related Eye Disease Study [10]. In addition, retro-illuminated photographs of the lens were obtained (Neitz CT-R camera; Neitz Instruments Co., Tokyo, Japan), and the percentage of the areas with cortical and posterior subcapsular lens opacities was measured using a grid. The standard to diagnose a nuclear cataract was a nuclear cataract grade of 4 or more, the standard to diagnose a posterior subcapsular cataract was a posterior subcapsular opacity amount of 0.01 or more, and the standard to diagnose a cortical cataract was a cortical opacity amount of 0.05 or more. The degree of fundus tessellation, defined as the visibility of the large choroidal vessels, was assessed using the fundus photographs of the macula and optic disc as described in detail previously [8]. It was graded using a scale that ranged from “0” for “no tessellation” to”3” for “marked tessellation”. Diabetic retinopathy was assessed using the fundus photographs and the Early Treatment of Diabetic Retinopathy Study (ETDRS) criteria. The minimum criterion for the diagnosis of diabetic retinopathy was the presence of at least one microaneurysm. The diagnosis for each individual was based on the grading of the eye with the highest stage of diabetic retinopathy. Glaucomatous optic neuropathy was defined using the criteria of the International Society of Geographic and Epidemiological Ophthalmology (ISGEO) [11]. Central serous choroidopathy was characterized by a serous retinal detachment in the macular region without hemorrhaging and without marked drusen on both fundus photographs and OCT images. Pseudoexfoliation was assessed by an experienced ophthalmologist during slit-lamp-assisted biomicroscopy of the anterior segment after pupillary dilation. The diagnosis of pseudoexfoliation was definite if the lens surface showed a central whitish coating with a diameter of little less than the normal pupillary diameter or if the periphery of the lens surface showed a whitish coating that was anteriorly bordered by a darker ring-like region on the lens surface. The assessment of pseudoexfoliation was performed only in phakic eyes. For the diagnosis of age-related macular degeneration, the International ARM (Age-Related Maculopathy Epidemiological Study Group) grading system was used.

Arterial hypertension was defined as systolic blood pressure ≥140 mm Hg and/or diastolic blood pressure ≥90 mm Hg and/or self-reported current treatment for arterial hypertension with antihypertensive medication. Diabetes mellitus was characterized by a blood glucose concentration ≥7.0 mmol/L, an HbA1c value ≥6%, a self-reported history of physician diagnosis of diabetes mellitus, or a history of drug treatments for diabetes (insulin or oral hypoglycemic agents). Body height was determined in a standardized manner with the shoes routinely removed. The subjects were asked to stand as upright as possible with the head raised as upright as possible. We used a stadiometer as the measuring instrument. Depressive symptoms were evaluated using a Chinese depression scale adapted from the Zung Self-Rating Depression Scale [12]. The Chinese depression scale used in our study has been validated previously [13,14]. The cerebrospinal fluid pressure (CSFP) was estimated as described in detail previously [15,16]. CSFP was best described by the following formula: Estimated CSFP [mmHg] = 0.44 x Body Mass Index [kg/m^2^] ± 0.16 x Diastolic Blood Pressure [mmHg]– 0.18 x Age [Years]—1.91. Based on an adaptation of the “Modification of Diet in Renal Disease (MDRD)” equation on the basis of data from Chinese chronic kidney disease patients, the estimated glomerular filtration rate (eGFR) was calculated as “eGFR_MDRD_ = 175 x (Serum Creatinine Concentration (mg/dL))^−1.234^ x Age (Years)^-0.179^ [if female, x 0.79]”, and reduced renal function was defined as an eGFR of less than 60 mL/min per 1·73 m^2^. Quality of life was assessed by standardized questions on mobility (“I have no problems in walking about; I have some problems in walking about; I am confined to bed”), self-care (“I have no problems with self-care; I have some problems washing or dressing myself; I am unable to wash or dress myself”), the performance of usual daily activities (e.g., work, study, housework, family or leisure activities) ("I have no problems with performing my usual daily activities; I have some problems with performing my usual daily activities; I am unable to perform my usual daily activities”), the presence of pain/discomfort (“I have no pain or discomfort; I have moderate pain or discomfort; I have extreme pain or discomfort”), and the presence of anxiety/depression (“I am not anxious or depressed; I am moderately anxious or depressed; I am extremely anxious or depressed). The total quality of life score was the sum of the replies to these 5 questions, and a higher score indicated a lower quality of life.

Statistical analysis was performed using a commercially available statistical software package (SPSS for Windows, version 22.0, IBM-SPSS, Chicago, IL, USA). To avoid a bias effect induced by a redundancy in the data from the same study participant, only one eye (right eye) of each study participant was included in the statistical analysis. Another reason to include only the data of right of eye in the statistical analysis was that some examinations (i.e., choroidal thickness (measurements) had been performed only for the right eyes of the study participants. As a first step, we examined the mean values (presented as the mean ± standard deviation) of the main outcome parameter, i.e., the self-rated depression score. As a second step, we performed a linear regression analysis in a univariate manner followed by a multivariate analysis, with the self-rated depression score as the dependent parameter. The multivariate analysis included as independent parameters all those variables that were correlated (*P*<0.10) with the self-rated depression score in the univariate analysis. We used a cut-off *P*-value value of <0.10 for inclusion into the multivariate analysis to avoid neglecting a parameter that would have been significantly associated with the outcome parameter in a multivariate analysis but, due to inter-dependencies with other parameters, was only marginally (i.e., *P*-value between 0.05 and 0.10) associated with the outcome parameter in the univariate analysis. We then eliminated all of the parameters that were no longer significantly associated with the self-rated depression score. As a last step, we re-added the prevalence of the main ocular disorders (if they had previously been taken out of the list of independent parameters) as single parameters to the multivariate model to re-test the potential associations between ocular disorders and self-rated depression in the final model. We calculated the standardized correlation coefficient beta, the non-standardized correlation coefficient B, and the 95% confidence intervals (CI). The prevalence of self-rated depression was described by frequency and 95%CI. Its associations with other parameters were examined through binary regression analysis with the calculation of odds ratios (ORs). All *P*-values were two-sided and considered statistically significant if the values were less than 0.05.

The datasets generated during and/or analyzed during the current study are available from the corresponding author on reasonable request.

## Results

Out of 3468 study participants, 3267 (94.2%) individuals participated in the interview assessing depression and answered all questions (S1 Dataset). The group of individuals participating compared to the group of individuals without complete answers in the interview was significantly younger (64.5 ± 9.7 years versus 67.3 ± 11.3 years; *P* = 0.001) and came significantly more often from the urban region than from the rural area (rural / urban region of habitation: 1481 / 1786 versus 152 / 49; *P*<0.001), while the differences in gender (men / women: 1419 / 1848 versus 86 / 115; *P* = 0.88), axial length (23.3 ± 1.1 mm versus 23.3 ± 1.2 mm; *P* = 0.99) and refractive error (-0.22 ± 2.12 diopters versus -0.38 ± 2.08 diopters; *P* = 0.44) were not statistically significant.

The mean self-rated depression score was 25.0 ± 5.9 (median: 23.3; range: 20–64). Self-rated depression defined as a self-rated depression score of >44 was present in 66 individuals (2.0%; 95%CI: 1.5, 2.5), and a self-rated depression score ≥59 was present in 5 individuals (0.2%; 95%CI: 0.02, 0.3).

In univariate analysis, a higher self-rated depression score was associated with the systemic parameters of older age (*P* = 0.06), female gender (*P*<0.001), rural region of habitation (*P* = 0.01), lower body mass index (*P* = 0.05), lower level of education (*P*<0.001), lower level of profession, lower cognitive score (*P*<0.001), less consumption of alcohol (*P*<0.001), lower physical activity (*P*<0.05), lower quality of life (*P*<0.001), higher blood concentration of high-density lipoproteins (*P*<0.001) and cholesterol (*P* = 0.03), lower blood concentration of triglycerides (*P* = 0.03), higher prevalence of diabetes mellitus (*P* = 0.04) and arterial hypertension (*P*<0.001), and lower estimated cerebrospinal fluid pressure (*P* = 0.07) as well as the ocular parameters of shorter axial length (*P*<0.001), shorter anterior corneal curvature radius (*P*<0.001), smaller anterior chamber depth (*P* = 0.03), lower intraocular pressure (*P* = 0.0.06), thinner retinal nerve fiber layer (*P* = 0.08), dry eye feeling (*P*<0.001), higher prevalence of keratoconus (*P*<0.001), glaucoma (*P* = 0.009), age-related macular degeneration (*P* = 0.01) and myopic retinopathy (*P* = 0.03) (Table 1). A higher self-rated depression score was not significantly associated (*P*>0.10) with smoking, blood concentration of glucose or low-density lipoproteins, systolic or diastolic blood pressure, estimated glomerular filtration rate, central corneal thickness, lens thickness, subfoveal choroidal thickness, macular retinal thickness, area of the optic disc, neuroretinal rim or parapapillary beta / gamma zone, prevalence of nuclear, cortical or subcapsular posterior cataract, diabetic retinopathy, retinal vein occlusions or central serous choroidopathy (Table 1).

**Table 1:** Univariate associations between self-rated depression scores and systemic and ocular parameters in the Beijing Eye Study.

Parameter	*P*-Value	Standardized Regression Coefficient beta	Non- Standardized Regression Coefficient B	95% Confidence Interval
Systemic Parameters				
Age (Years)	0.056	0.03	0.02	0.00, 0.04
Gender (Men / Women)	<0.001	0.13	1.56	1.15, 1.96
Rural / Urban Region of Habitation	0.011	-0.11	-1.29	-1.69, -0.89
Body Mass Index (kg/m^2^)	0.05	-0.03	-0.05	-0.10, 0.00
Level of Education (1–5)	<0.001	-0.17	-0.92	-1.11, -0.73
Self-Reported Income	<0.001	0.10	2.28	1.53, 3.04
Profession (No Occupation / Worker / Peasant / Intellectual / Business Person / Military)	<0.001	-0.12	-0.49	-0.65, -0.33
Cognitive Score	<0.001	-0.17	-0.27	-0.33, -0.21
Alcohol Consumption Quantity	<0.001	-0.07	-0.33	-0.49, -0.16
Alcohol Consumption Frequency	<0.001	-0.07	-0.23	-0.34, -0.11
Smoking Never / Former / Current	0.19	-0.02	-0.17	-0.42, 0.08
Smoking Never / Ever	0.06	-0.03	-0.42	-0.85, 0.02
Smoking Package Years	0.98	0.00	0.00	-0.01, 0.01
Smoking, Present Quantity	0.93	-0.002	-0.01	-0.27, 0.25
Physical Activity				
“How Many Days Do You Walk?”	0.03	-0.04	-0.10	-0.18, -0.01
“How Many Days Do You Do Vigorously Intensive Sports or Activities?”	0.37	-0.02	-0.08	-0.24, 0.09
“How Many Days Do You Do Moderately Intensive Sports or Activities?”	0.01	-0.05	-0.08	-0.15, -0.02
“How Many Hours Do You Sit Per Day?”	0.30	-0.02	-0.04	-0.12, 0.04
Quality of Life				
Summed Score	<0.001	0.42		
Mobility: I have no / some problems in walking about / I am confined to bed	<0.001	0.25	4.90	4.23, 5.56
Self-Care: I have no / some problems in washing or dressing myself / I am unable to wash or dress myself	<0.001	0.19	4.66	3.81, 5.51
Usual Activities (e.g., work, study, housework, family or leisure activities): I am able to perform my usual activities / I have some problems with performing my usual activities / I am unable to perform my usual activities	<0.001	0.25	5.36	4.65, 6.09
Pain/Discomfort:	<0.001	0.20	2.42	2.01, 2.83
Anxiety/Depression: I am not / moderately / extremely anxious or depressed	<0.001	0.52	9.51	8.97, 10.0
Blood Concentration of:				
Glucose (mmol/L)	0.68	0.68	0.03	-0.12, 0.19
High-Density Lipoproteins (mmol/L)	<0.001	0.10	1.30	0.77, 1.82
Low-Density Lipoproteins (mmol/L)	0.23	0.03	0.16	-0.10, 0.42
Triglycerides (mmol/L)	0.03	-0.05	-0.23	-0.43, -0.03
Cholesterol (mmol/L)	0.03	0.04	0.27	0.02, 0.51
Diabetes Mellitus, Prevalence	0.04	0.04	0.59	0.02, 1.17
Systolic Blood Pressure (mmHg)	0.10	0.03	0.01	0.00, 0.02
Diastolic Blood Pressure (mmHg)	0.95	0.001	0.001	-0.02, 0.02
Mean Blood Pressure (mmHg)	0.40	0.02	0.01	-0.01, 0.02
Arterial Hypertension	<0.001	0.07	0.79	0.39, 1.20
Estimated Cerebrospinal Fluid Pressure (mm Hg)	0.07	-0.03	-0.05	-0.10, 0.003
Estimated Glomerular Filtration Rate (GFR) (mL/min / 1·73 m^2^) (MDRD Formula)	0.73	0.01	0.003	-0.01, 0.02
Estimated Glomerular Filtration Rate (mL/min / 1·73 m^2^) (CKDE Formula)	0.20	-0.03	-0.02	-0.04, 0.01
Chronic Kidney Disease, Prevalence (GFR<60 mL/min / 1·73 m^2^) (MDRD Formula)	0.30	0.03	1.10	-1.00, 3.19
Chronic Kidney Disease, Prevalence (GFR<60 mL/min / 1·73 m^2^) (CKDE Formula)	0.23	0.02	0.98	-0.61, 2.56
Ocular Parameters				
Refractive Error (Diopters)	-------			
Axial Length (mm)	<0.001	-0.08	-0.42	-0.61, -0.24
Anterior Corneal Curvature Radius (mm)	<0.001	-0.09	-1.96	-2.76, -1.15
Central Corneal Thickness (μm)	0.14	-0.03	-0.01	-0.01, 0.00
Anterior Chamber Depth (mm)	0.03	-0.04	-0.46	-0.88, -0.04
Lens Thickness (mm)	0.47	0.01	0.23	-0.39, 0.85
Intraocular Pressure (mmHg)	0.06	-0.03	-0.07	-0.14, 0.00
Retinal Nerve Fiber Layer Thickness (μm)	0.08	-0.03	-0.01	-0.03, 0.00
Subfoveal Choroidal Thickness (μm)	0.95	0.001	0.00	-0.002, 0.002
Macular Retinal Thickness (μm)	0.23	-0.03	-0.005	-0.01, 0.003
Optic Disc Size (mm^2^)	0.99	0.00	-0.001	-0.46, 0.46
Neuroretinal Rim Area (mm^2^)	0.90	0.003	0.04	-0.63, 0.71
Parapapillary Beta Zone (mm^2^)	0.93	-0.002	-0.01	-0.22, 0.20
Parapapillary Gamma Zone (mm^2^)	0.96	0.002	0.00	-0.002, 0.002
Dry Eye, Yes or No	<0.001	0.08	0.94	0.54, 0.1.35
Dry Eye, Number of Days	<0.001	0.10	0.21	0.14, 0.29
Keratoconus (Anterior Corneal Curvature Refractive Power ≥48 Diopters)	0.009	0.05	2.98	0.73, 5.22
Keratoconus (Anterior Corneal Curvature Refractive Power ≥49 Diopters)	<0.001	0.08	10.2	5.52, 14.8
Nuclear Cataract	0.03	0.04	0.52	0.06, 0.98
Cortical Cataract	0.12	0.03	0.47	-0.13, 1.06
Subcapsular Posterior Cataract	0.21	0.03	0.63	-0.34, 1.59
Glaucoma, Prevalence, Total	0.009	0.05	1.30	0.32, 2.28
Open-Angle Glaucoma	0.10	0.03	1.00	-0.19, 2.18
Primary Angle-Closure Glaucoma	0.05	0.03	1.74	0.003, 3.47
Age-Related Macular Degeneration, Prevalence, Total	0.01	0.04	0.57	0.12, 1.03
Age-Related Macular Degeneration, Early Stage	0.50	0.01	0.26	-0.48, 0.99
Age-Related Macular Degeneration, Intermediate Stage	0.006	0.05	0.74	0.21, 1.28
Age-Related Macular Degeneration, Late Stage	0.10	-0.03	-1.93	-4.24, 0.39
Diabetic Retinopathy, Prevalence	0.92	0.002	0.07	-1.23, 1.36
Diabetic Retinopathy, Score	0.95	0.001	0.04	-1.27, 1.34
Retinal Vein Occlusion, Total	0.18	-0.02	-1.13	-2.77, -0.52
Central Retinal Vein Occlusion	0.25	-0.02	-3.01	-8.13, 2.12
Branch Retinal Vein Occlusion	0.30	-0.02	-0.91	-2.65, 0.82
Central Serous Choroidopathy	0.30	-0.02	-0.09	-0.26, 0.08
Myopic Retinopathy	0.03	0.04	1.85	0.19, 3.50

The first step in the multivariate analysis assessed the self-rated depression score as the dependent variable and all the systemic parameters for which the *P*-value of their univariate association with the self-rated depression score was <0.10 as independent variables. We then eliminated profession (*P* = 0.92), quantity of alcohol consumption (*P* = 0.92), blood concentration of high-density lipoproteins (*P* = 0.83), history of ever smoking (*P* = 0.85), number of days with vigorous sport activities (*P* = 0.61), level of education (*P* = 0.74), self-reported income (*P* = 0.38), frequency of alcohol consumption (*P* = 0.31), presence of diabetes mellitus (*P* = 0.4), systolic blood pressure (*P* = 0.09), and number of days with walking (*P* = 0. 06). In the resulting model, higher self-rated depression scores were associated with younger age, male gender, rural region of habitation, lower body mass index, lower cognitive function score, lower quality of life, lower blood concentration of triglycerides, higher blood concentration of cholesterol, and the presence of arterial hypertension (Table 2).

**Table 2:** Associations (multivariate analysis) between the self-rated depression score and systemic variables in the Beijing Eye Study.

Parameter	*P*-Value	Standardized Regression Coefficient beta	Non- Standardized Regression Coefficient B	95% Confidence Interval	Variance Inflation Factor
Age (Years)	0.003	-0.07	-0.04	-0.07, -0.01	1.38
Gender (Men / Women)	0.003	0.06	0.71	0.25, 1.18	1.10
Rural / Urban Region of Habitation	<0.001	-0.08	-0.99	-1.48, -0.50	1.23
Body Mass Index (kg/m^2^)	<0.001	-0.12	-0.19	-0.25, -0.12	1.20
Cognitive Function Score	<0.001	-0.08	-0.13	-0.19, -0.06	1.18
Quality of Life Total Score	<0.001	0.41	2.38	2.16, 2.61	1.07
Triglycerides Concentration (mmol/L)	0.009	-0.05	-0.27	-0.46, -0.07	1.17
Cholesterol Concentration (mmol/L)	0.02	0.05	0.29	0.05, 0.54	1.19
Arterial Hypertension	0.02	0.05	0.55	0.08, 1.02	1.14

In the second step, we added all ocular parameters that were significantly associated with the self-rated depression score in the univariate analysis to the model. Due to a lack of significance, we then eliminated arterial hypertension (*P* = 0.91), retinal nerve fiber layer thickness (*P* = 0.80), myopic maculopathy (*P* = 0.93), any stage of age-related macular degeneration (*P* = 0.79), the prevalence of angle-closure glaucoma (*P* = 0.63) and open-angle glaucoma (*P* = 0.93), axial length (*P* = 0.81), the prevalence of keratoconus (defined as corneal refractive power ≥48 diopters) (*P* = 0.79), late stage age-related macular degeneration (*P* = 0.75), overall glaucoma (*P* = 0.50), anterior chamber depth (*P* = 0.45), intraocular pressure (*P* = 0.48), the prevalence of nuclear cataract (*P* = 0.40), dry eye feeling (*P* = 0.41), keratoconus (defined as corneal refractive power ≥49 diopters) (*P* = 0.18), intermediate stage age-related macular degeneration (*P* = 0.14), and the blood concentration of cholesterol (*P* = 0.05). In the final model, higher self-rated depression scores were significantly (regression coefficient r^2^: 0.22) associated with a higher number of days with dry eye feeling (*P*<0.001; beta: 0.09; B: 0.20; 95%CI: 0.12, 0.29) and a shorter corneal curvature radius (*P* = 0.03; beta: -0.04; B: 1.01; 95%CI: -1.90, -0.12), after adjusting for age, gender, region of habitation, body mass index, cognitive function score, life quality score and blood concentration of triglycerides (Table 3). If the corneal curvature radius factor was eliminated and replaced by the presence of keratoconus, the latter was not significantly (P = 0.41) associated with self-rated depression scores.

**Table 3:** Associations (multivariate analysis) between the self-rated depression score and systemic and ocular variables in the Beijing Eye Study.

Parameter	*P*-Value	Standardized Regression Coefficient beta	Non- Standardized Regression Coefficient B	95% Confidence Interval	Variance Inflation Factor
Systemic Parameters					
Age (Years)	0.005	-0.06	-0.04	-0.07, -0.01	1.30
Gender (Men / Women)	0.02	0.05	0.55	0.08, 1.01	1.11
Rural / Urban Region of Habitation	<0.001	-0.10	-1.17	-1.67, -0.68	1.26
Body Mass Index (kg/m^2^)	<0.001	-0.10	-0.15	-0.21, -0.10	1.14
Cognitive Function Score	<0.001	-0.07	-0.12	-0.18, -0.05	1.14
Quality of Life (Inverse Score)	<0.001	0.41	2.39	2.16, 2.62	1.17
Blood Concentration Triglycerides (mmol/L)	0.02	-0.05	-0.22	-0.41, -0.03	1.05
Ocular Parameters					
Anterior Corneal Curvature Radius (mm)	0.03	-0.04	-1.01	-1.90, -0.12	1.07
Number of Days with Dry Eye Feeling	<0.001	0.09	0.20	0.12, 0.29	1.07

Adding the prevalence of the diseases age-related macular degeneration (*P* = 0.10), glaucoma (*P* = 0.77), diabetic retinopathy (*P* = 0.77), nuclear cataract (*P* = 0.35), cortical cataract (*P* = 0.58) or posterior subcapsular cataract (*P* = 0.28) as single parameters to the model revealed no significant correlations between these factors and self-rated depression scores. Adding best corrected visual acuity (measured in logMAR (negative decadic logarithm of the minimum angle of resolution)) to the model showed a marginally significant association with self-rated depression scores (*P* = 0.05; beta: 0.04; B: 1.56; 95%CI: -0.01, 3.13).

If self-rated depression was defined as a self-rated depression score higher than 44, its prevalence, as examined in a binary regression analysis, was associated only with lower cognitive function (*P*<0.001; OR: 0.92; 95%CI: 0.87, 0.96) and lower quality of life (*P*<0.001; OR: 2.11; 95%CI: 1.83, 2.44). Adding the diseases age-related macular degeneration (*P* = 0.14), glaucoma (*P* = 0.72), diabetic retinopathy (*P* = 0.39), nuclear cataract (*P* = 0.83), cortical cataract (*P* = 0.21) or posterior subcapsular cataract (*P* = 0.99) as single parameters to the model revealed no significant correlations between these parameters and the occurrence of self-rated depression. Adding visual acuity (logMAR) to the model also did not reveal a significant association (*P* = 0.10).

## Discussion

In our population-based study on a population aged 50+ years in Greater Beijing, the prevalence of self-rated depression (defined by a self-rated depression score >44) was 2.0% (95%CI: 1.5, 2.5). A higher self-rated depression score was significantly associated with a higher number of days with dry eye feeling and shorter corneal curvature radius after adjusting for age, gender, region of habitation, body mass index, cognitive function score, life quality score and blood concentration of triglycerides. None of the major ocular diseases, that is, age-related macular degeneration, glaucoma, diabetic retinopathy, nuclear cataract, cortical cataract or posterior subcapsular cataract, was significantly associated with the self-rated depression score. Lower best corrected visual acuity showed a marginally significant association with higher self-rated depression scores (*P* = 0.05).

The result of an association between dry eye symptoms and a higher prevalence of depressive symptoms confirmed previous studies. In a population-based cross-sectional study, Kim and coworkers examined 657 Korean individuals aged 65+ years and reported that depression (assessed by the Korean version of the Short Geriatric Depression Scale) was associated with dry eye symptoms in subjects with normal or mildly reduced tear production [3]. Similar findings were reported by Hallak and colleagues, who examined 53 patients with dry eye symptoms and 41 controls and found that after adjusting for age, sex, race, and psychiatric medication, dry eye symptoms and depressive symptoms were significantly correlated [4]. In a previous study on the participants of the Beijing Eye Study 2006, individuals with dry eye symptoms showed a higher prevalence of self-rated depression [5]. In a systematic review and meta-analysis, Wan et al. found that depression scores and anxiety scores were higher in patients with dry eye symptoms than in controls [6]. In a recent investigation carried out by Mrugacz and colleagues, the concentrations of the pro-inflammatory cytokines IL-17 and TNF-α in tears were associated with the clinical severity of dry eye disease in patients with depression [7]. In particular, the latter study suggested a pathogenetic association between depression and dry eye through tear composition since dry eye has been described to be associated with, or even caused by, a subclinical inflammation of the external eye including the conjunctiva. Studies by Pflugfelder and others have shown that the concentrations of inflammatory cytokines such as IL-1a, IL-6, IL-8 and TNF-α are increased in the conjunctival epithelium of patients with Sjögren syndrome, which is characterized by severe dry eye symptoms [17]. Additionally, HLA-DR antigen expression in the conjunctival epithelium is elevated in patients with dry eye disease, as shown by flow cytometric examinations. In a similar manner, Solomon and colleagues found that the concentrations of IL-1a and IL-1b were increased in tear fluid in the tear fluid and conjunctiva of patients with dry eye disease [18]. The production of inflammatory cytokines can also be influenced by antidepressant drugs. Munzer and associates reported that the antidepressant drug citalopram increased the production of IL-1b, IL-6, IL-22 and TNF-α, the antidepressant mitrazepine increased the production of IL-1b, IL-22 and TNF-α, and the antidepressant escitalopram decreased the production of IL-17 [19]. These results fit with the notion that the association between depression and dry eye is at least partially caused by changes in the ocular surface and may reflect a localized phenomenon [7]. In addition, an anticholinergic effect of anti-depressant medication has been discussed as being connected with a dry eye feeling [20,21]. Most participants in our study, however, did not take any antidepressant medication, so the dry eye might not have been a side-effect of systemically administered anti-depressant medication. An additional possibility to explain the association between depression and dry eye may be that the symptoms of dry eye syndrome, such as pain, could induce the occurrence of anxiety and depressive symptoms in susceptible individuals.

The findings obtained in our study regarding a lack of associations between depression and other ocular disorders contradict previous investigations. Augustin and colleagues examined 120 patients with advanced age-related macular degeneration in third-referral centers [22]. As assessed by the Hospital Anxiety and Depression Scale, self-rated depression was associated with lower visual acuity and indirectly associated with a higher stage of age-related macular degeneration. As a side observation in a randomized clinical trial, Brody et al. found depressive disorders in 49 (33%) individuals among 151 community-dwelling adults with advanced age-related macular degeneration [23]. This rate was twice as high as the rate observed in the community-dwelling elderly. In another hospital-based study, Lee and colleagues examined 107 patients with age-related macular degeneration treated with intravitreal ranibizumab and reported that the prevalence of depression was 26%, with a positive association with older age, lower best corrected visual acuity, a longer duration of the disease and a higher number of previous treatments for the disorder [24]. Similar results were reported by Jivrai et al. and by Popescu and colleagues [25,26]. In a meta-analysis of published studies, Dawson et al. reported that patients with age-related macular degeneration were more likely to show symptoms of depression than of anxiety [27]. The meta-analysis, however, did not allow the drawing of conclusions of whether age-related macular degeneration was associated with an increased level of depression. An increased level of depression was also found in patients with ocular inflammatory disease and with decreased self-reported visual function loss in general [28,29]. Associations between decreased vision and depression were examined by van der Aa and colleagues [30]. They performed telephone interviews with 615 visually impaired older adults aged 60+ years from outpatient low-vision rehabilitation centers and carried out face-to-face interviews with 1232 community-dwelling normally sighted peers. The prevalence of major depressive disorder (5.4%) and anxiety disorders (7.5%) and the prevalence of subthreshold depression (32.2%) and subthreshold anxiety (15.6%) were significantly higher in the visually impaired older adults than in the normally sighted individuals. Correlations between depression and glaucoma were assessed by Wang et al [31]. They reported that among 6760 participants of the National Health and Nutrition Examination Survey aged 40+ years, the presence of self-reported glaucoma was significantly associated with depression after adjusting for demographic factors, while this association was not statistically significant after adjusting for self-reported general health conditions. Self-reported measures of visual function were significantly associated with depression. Associations between glaucoma and depression were also reported by Skalicky and Goldberg and Zhou et al., among others [32–37]. In a recent meta-analysis, Zheng and colleagues reported an overall pooled prevalence of depression or depressive symptoms with eye disease of 25% [38]. The highest prevalence of depression was found for dry eye disease at 29%, followed by 25% for glaucoma patients, 24% for age-related macular degeneration patients, and 23% for cataract patients.

The reasons for the discrepancies between these previous investigations and our study with respect to the lack of associations between self-rated depression and the prevalence of major ocular disorders remain unclear. Most of these studies had a hospital-based recruitment of study participants, included a relatively small number of study participants; not all of the previous studies had a control group; and if a multivariate analysis was performed, it may not have included visual loss or all major non-ocular factors influencing the prevalence of self-rated depression.

The self-rated depression prevalence of 2.0% (95%CI: 1.5, 2.5) in our study population agrees with the results of a meta-analysis that included 17 studies from China, in which the current, 12-month and lifetime prevalences of major depressive disorder were 1.6, 2.3, 3.3%, respectively [39]. As observed for self-rated depression in our study, the prevalence of current major depressive disorder in the meta-analysis was higher in rural areas than in urban regions (2.0 versus 1.7%), and it was higher in women than in men (2.1 versus 1.3%). It should however be taken into account we assessed a self-rated depression score and that we did not assess the prevalence of “current major depressive disorder”.

When discussing the results of our study, its limitations have to be taken into account. First, the data on self-rated depression depended on the self-assessment by the study participants in the face-to-face interviews. Since psychiatric disorders have a negative perception in China, the prevalence of depression based on self-reported data may be underestimated. This limitation may be valid for all societies but may be more prevalent in East Asian cultures. The figures on the prevalence of depression reported in previous studies from China are however in agreement with the data on the prevalence of self-rated depression found in our study population and supported the findings of the present investigation [27]. Second, and in a parallel manner, a diagnosis of a depressive disorder is best determined in a face-to-face interview conducted by a specialist in the field. However, although self-ratings of depression are subjective and suboptimal, they have been shown to be valuable proxies for expert ratings [40,41]. In addition, the diagnosis retrieved from Zung’s self-rating scale as applied in the present study has previously been demonstrated to correlate well with expert ratings [42,43]. Since however a diagnosis of depression could not be based solely on a cut-off value of a self-rated depressive score, most of the statistical analysis performed in our study applied a continuous analysis of the depression score and not a binary regression analysis of the prevalence of self-rated depression. The self-rated depression score assessing depressive symptomatology has been widely used also in other epidemiological studies. In these studies, co-morbidities between the depression symptomatology and somatic disorders such as cardiovascular disorders have been observed for expert ratings as well as for self-ratings [44,45]. In addition, recent genetic analyses indicated that the underlying mechanisms were largely shared between major depressive disorder and current depressive symptoms, underlying the value of even very economic assessments such as the one carried out in this study [46]. Third, the study population with an age of 50+ years had experienced major societal changes and economic developments in their lifetime. This elderly generation may differ from the young generations in China and from populations in other countries, especially with respect to a disorder such as depression, which may particularly be influenced by changes in general life conditions. It therefore remains unclear the extent to which the results of the present study can be transferred to other populations. Fourth, as for any population-based study, the non-participation of eligible individuals may lead to a bias. The general participation rate in the Beijing Eye Study was, however, 78.8%, and 94.2% of the participants in the Beijing Eye Study answered all questions referring to depression. These figures may have provided a sufficient basis for the statistical analysis of the measurements. Fifth, clinical tests for the evaluation of dry eye feeling might have been added to the study to objectively assess the diagnosis of “dry eye”. These tests, such the measurement of the tear-film break-up time, the assessment of fluorescein staining of the cornea, the examination of Meibomian gland dysfunction and Schirmer´s test, were previously included in a test series [2]. The dry eye symptoms as reported subjectively by the study participants were, however, not significantly associated with the results of any of these tests [47]. The present study therefore did not include examinations of tear film stability, corneal surface integrity or tear volume.

In conclusion, dry eye feeling was the only common ocular disorder associated with increased self-rated depression scores in a multivariate analysis, while the occurrence of age-related macular degeneration, any type of glaucoma, diabetic retinopathy, any type of cataract and keratoconus were not significantly associated with increased self-rated depression scores. Lower visual acuity was marginally associated. The prevalence of self-rated depression in the population aged 50+ years in Greater Beijing was 2.0% (95%CI: 1.5, 2.5).

## Supporting information

S1 Dataset Datafile containing the microdata as basis of this study. (SAV)

## References

[1] Global, regional, and national incidence, prevalence, and years lived with disability for 310 diseases and injuries, 1990–2015: a systematic analysis for the Global Burden of Disease Study 2015. Lancet. 2016;388: 1545–1602. 10.1016/S0140-6736(16)31678-6 27733282 PMC5055577

[2] Jie Y, Xu L, Wu YY, Jonas JB. Prevalence of dry eye among adult Chinese in the Beijing Eye Study. Eye. 2009;23: 688–693. 10.1038/sj.eye.6703101 18309341

[3] Kim KW, Han SB, Han ER, Woo SJ, Lee JJ, Yoon JC, et al Association between depression and dry eye disease in an elderly population. Invest Ophthalmol Vis Sci. 2011;52: 7954–7958. 10.1167/iovs.11-8050 21896858

[4] Hallak JA, Tibrewal S, Jain S. Depressive symptoms in patients with dry eye disease: a case-control study using the Beck Depression Inventory. Cornea. 2015;34: 1545–1550. 26426334 10.1097/ICO.0000000000000641 PMC4636920

[5] Labbé A, Wang YX, Jie Y, Baudouin C, Jonas JB, Xu L. Depression and dry eye disease. The Beijing Eye Study. Br J Ophthalmol. 2013;97: 1399–1403. 24013959 10.1136/bjophthalmol-2013-303838

[6] Wan KH, Chen LJ, Young AL. Depression and anxiety in dry eye disease: a systematic review and meta-analysis. Eye (Lond). 2016;30: 1558–1567. 27518547 10.1038/eye.2016.186 PMC5177754

[7] Mrugacz M, Ostrowska L, Bryl A, Szulc A, Zelazowska-Rutkowska B, Mrugacz G. Pro-inflammatory cytokines associated with clinical severity of dry eye disease of patients with depression. Adv. Med. Sci. 2017;62: 338–344. 10.1016/j.advms.2017.03.003 28511072

[8] Yan YN, Wang YX, Xu L, Xu J, Wei WB, Jonas JB. Fundus tessellation: Prevalence and associated factors. The Beijing Eye Study 2011. Ophthalmology. 2015;122: 1873–1880. 10.1016/j.ophtha.2015.05.031 26119000

[9] Xu J, Xu L, Du KF, Shao L, Chen CX, Zhou JQ, et al Subfoveal choroidal thickness in diabetes and diabetic retinopathy. The Beijing Eye Study 2011. Ophthalmology. 2013;120: 2023–2028. 10.1016/j.ophtha.2013.03.009 23697958

[10] Age-Related Eye Disease Study Research Group. The Age-Related Eye Disease Study (AREDS) system for classifying cataracts from photographs: AREDS report no. 4. Am J Ophthalmol. 2001;131: 167–175. 11228291 10.1016/s0002-9394(00)00732-7 PMC2032014

[11] Foster PJ, Buhrmann R, Quigley HA, Johnson GJ. The definition and classification of glaucoma in prevalence surveys. Br J Ophthalmol. 2002;86: 238–242. 11815354 10.1136/bjo.86.2.238 PMC1771026

[12] Zung WW A Self-rating depression scale. Arch Gen Psychiatry. 1965;12: 63–70. 14221692 10.1001/archpsyc.1965.01720310065008

[13] Shen M, Hu M, Sun Z. Development and validation of brief scales to measure emotional and behavioural problems among Chinese adolescents. BMJ Open. 2017;7: e012961 10.1136/bmjopen-2016-012961 28062469 PMC5223728

[14] Lee HC, Chiu HF, Wing YK, Leung CM, Kwong PK, Chung DW. The Zung Self-rating Depression Scale: screening for depression among the Hong Kong Chinese elderly. J. Geriatr. Psychiatry Neurol. 1994;7: 216–220. 10.1177/089198879400700404 7826489

[15] Xie XB, Zhang XJ, Fu J, Wang H, Jonas JB, Peng XX, et al Intracranial pressure estimation by orbital subarachnoid space measurement. Crit Care. 2013;17: R162 10.1186/cc12841 23883736 PMC4056099

[16] Jonas JB, Nangia V, Wang N, Bhate K, Nangia P, Nangia P, et al Trans-lamina cribrosa pressure difference and open-angle glaucoma. The central India eye and medical study. PLoS One. 2013;8: e8228. 10.1371/journal.pone.0082284 PMC3855749 24324767

[17] Pflugfelder SC, Jones D, Ji Z. Altered cytokine balance in the tear fluid and conjunctiva of patients with Sjogren’s syndrome keratoconjunctivitis sicca. Curr Eye Res. 1999;19: 201–211. 10487957 10.1076/ceyr.19.3.201.5309

[18] Solomon A, Dursun D, Liu Z. Pro- and anti-inflammatory form of interleukin-1 in the tear fluid and conjunctiva of patients with dry eye disease. Invest Ophthalmol Vis Sci. 2001;42: 2283–2292. 11527941

[19] Munzer A, Sack U, Mergl R, Schönherr J, Petersein C, Bartsch S, et al Impact of antidepressants on cytokine production of depressed patients in vitro. Toxins. 2013;5: 2227–2240. 10.3390/toxins5112227 24257035 PMC3847723

[20] Wang TJ, Wang IJ, Hu CC, Lin HC. Comorbidities of dry eye disease: a nationwide population-based study. Acta Ophthalmol. 2012;90: 663–668. 10.1111/j.1755-3768.2010.01993.x 20809911

[21] Enriquez de Salamanca A, Siemasko KF, Diebold Y. Expression of muscarinic and adrenergic receptors in n ormal human conjunctival epithelium. Invest Ophthalmol Vis Sci. 2005;46: 504–513. 10.1167/iovs.04-0665 15671275

[22] Augustin A, Sahel JA, Bandello F, Dardennes R, Maurel F, Negrini C, et al Anxiety and depression prevalence rates in age-related macular degeneration. Invest Ophthalmol Vis Sci. 2007;48: 1498–1503. 10.1167/iovs.06-0761 17389477

[23] Brody BL, Gamst AC, Williams RA, Smith AR, Lau PW, Dolnak D, et al Depression, visual acuity, comorbidity, and disability associated with age-related macular degeneration. Ophthalmology. 2001;108: 1893–900. 11581068 10.1016/s0161-6420(01)00754-0

[24] Lee WJ, Cho HY, Kim DH, Yu HG, Oh J, Kim JS, et al Depression of late age-related macular degeneration patients in Korea. Asia Pac J Ophthalmol (Phila). 2013;2: 23–27. 26107864 10.1097/APO.0b013e31827be8b1

[25] Jivraj J, Jivraj I, Tennant M, Rudnisky C. Prevalence and impact of depressive symptoms in patients with age-related macular degeneration. Can J Ophthalmol. 2013;48: 269–273. 10.1016/j.jcjo.2013.03.007 23931465

[26] Popescu ML, Boisjoly H, Schmaltz H, Kergoat MJ, Rousseau J, Moghadaszadeh S, et al Explaining the relationship between three eye diseases and depressive symptoms in older adults. Invest Ophthalmol Vis Sci. 2012;53: 2308–2313. 10.1167/iovs.11-9330 22427589

[27] Dawson SR, Mallen CD, Gouldstone MB, Yarham R, Mansell G. The prevalence of anxiety and depression in people with age-related macular degeneration: a systematic review of observational study data. BMC Ophthalmol. 2014;14: 78 10.1186/1471-2415-14-78 24923726 PMC4094542

[28] Qian Y, Glaser T, Esterberg E, Acharya NR. Depression and visual functioning in patients with ocular inflammatory disease. Am J Ophthalmol. 2012;153: 370–378. 10.1016/j.ajo.2011.06.028 21924399 PMC3243823

[29] Zhang X, Bullard KM, Cotch MF, Wilson MR, Rovner BW, McGwin G Jr, et al Association between depression and functional vision loss in persons 20 years of age or older in the United States, NHANES 2005–2008. JAMA Ophthalmol. 2013;131: 573–581. 10.1001/jamaophthalmol.2013.2597 23471505 PMC3772677

[30] van der Aa HP, Comijs HC, Penninx BW, van Rens GH, van Nispen RM. Major depressive and anxiety disorders in visually impaired older adults. Invest Ophthalmol Vis Sci. 2015;56: 849–854. 10.1167/iovs.14-15848 25604690

[31] Wang SY, Singh K, Lin SC. Prevalence and predictors of depression among participants with glaucoma in a nationally representative population sample. Am J Ophthalmol. 2012;154: 436–444.e2. 10.1016/j.ajo.2012.03.039 22789562 PMC3422443

[32] Skalicky S, Goldberg I. Depression and quality of life in patients with glaucoma: a cross-sectional analysis using the Geriatric Depression Scale-15, assessment of function related to vision, and the Glaucoma Quality of Life-15. J Glaucoma. 2008;17: 546–551. 10.1097/IJG.0b013e318163bdd1 18854731

[33] Zhou C, Qian S, Wu P, Qiu C. Anxiety and depression in Chinese patients with glaucoma: sociodemographic, clinical, and self-reported correlates. J Psychosom Res. 2013;75:75–82. 10.1016/j.jpsychores.2013.03.005 23751243

[34] Yochim BP, Mueller AE, Kane KD, Kahook MY. Prevalence of cognitive impairment, depression, and anxiety symptoms among older adults with glaucoma. J Glaucoma. 2012;21: 250–254. 10.1097/IJG.0b013e3182071b7e 21336151

[35] Mabuchi F, Yoshimura K, Kashiwagi K, Shioe K, Yamagata Z, Kanba S, et al High prevalence of anxiety and depression in patients with primary open-angle glaucoma. J Glaucoma. 2008;17: 552–557. 10.1097/IJG.0b013e31816299d4 18854732

[36] Lim NC, Fan CH, Yong MK, Wong EP, Yip LW. Assessment of depression, anxiety, and quality of life in Singaporean patients with glaucoma. J Glaucoma. 2016;25: 605–612. 10.1097/IJG.0000000000000393 26950574

[37] Zhang X, Olson DJ, Le P, Lin FC, Fleischman D, Davis RM. The association between glaucoma, anxiety and depression in a large population. Am J Ophthalmol. 2017;183: 37–41. 10.1016/j.ajo.2017.07.021 28760639

[38] Zheng Y, Wu X, Lin X, Lin H. The prevalence of depression and depressive symptoms among eye disease patients: a systematic review and meta-analysis. Sci Rep. 2017;7: 46453 10.1038/srep46453 28401923 PMC5388862

[39] Gu L, Xie J, Long J, Chen Q, Chen Q, Pan R, et al Epidemiology of major depressive disorder in mainland china: a systematic review. PLoS One. 2013;8: e65356 10.1371/journal.pone.0065356 23785419 PMC3681935

[40] Martin J, Streit F, Treutlein J, Lang M, Frank J, Forstner AJ, et al Expert and self-assessment of lifetime symptoms and diagnosis of major depressive disorder in large-scale genetic studies in the general population: comparison of a clinical interview and a self-administered checklist. Psychiatr Genet. 2017;27: 187–196. 10.1097/YPG.0000000000000182 28731911 PMC5576521

[41] Hilsenroth MJ, Baity MR, Mooney MA, Meyer GJ. DSM-IV Major Depressive Episode criteria: An evaluation of reliability and validity across three different rating methods. Int J Psychiatry Clin Pract. 2004;8: 3–10. 10.1080/13651500310004795 24937577

[42] Biggs JT, Wylie LT, Ziegler VE. Validity of the Zung Self-rating Depression Scale. Br J Psychiatry. 1978;132: 381–385. 638392 10.1192/bjp.132.4.381

[43] Kitamura T, Shima S, Sugawara M, Toda MA. Temporal variation of validity of self-rating questionnaires: repeated use of the General Health Questionnaire and Zung’s Self-rating Depression Scale among women during antenatal and postnatal periods. Acta Psychiatr Scand. 1994;90: 446–450. 7892778 10.1111/j.1600-0447.1994.tb01622.x

[44] Katon WJ. Clinical and health services relationships between major depression, depressive symptoms, and general medical illness. Biol Psychiatry. 2003;54: 216–226. 12893098 10.1016/s0006-3223(03)00273-7

[45] Cohen BE, Edmondson D, Kronish IM. State of the art review: depression, stress, anxiety, and cardiovascular disease. Am J Hypertens. 2015;28: 1295–302. 10.1093/ajh/hpv047 25911639 PMC4612342

[46] Wray NR, Ripke S, Mattheisen M, Trzaskowski M, Byrne EM, Abdellaoui A, et al Genome-wide association analyses identify 44 risk variants and refine the genetic architecture of major depression. Nat Genet. 2018;50: 668–681. 10.1038/s41588-018-0090-3 29700475 PMC5934326

[47] Schein OD, Muñoz B, Tielsch JM, Bandeen-Roche K, West S. Prevalence of dry eye among the elderly. Am J Ophthalmol. 1997;124: 723–728. 9402817 10.1016/s0002-9394(14)71688-5

